# Nuts and Cheese

**Published:** 1869-01

**Authors:** 


### NUTS AND CHEESE

Promote digestion as a general rule; the conditions being that the nuts should be ripe and the cheese old, both to be eaten at the close of dinner; the digesting agent in both is a peculiar oil which has the property of acting chemically on what has been eaten, and thus preparing it for being the more easily appropriated to the purposes of nutrition. Many think that the more solid portions of the nut should not be swallowed. This is an error; those particles of solid matter are not digested, it is true, but they are passed through the system unchanged, and act as a mechanical stimulant to the action of the internal organs, as white mustard-seed swallowed whole are known to do, thus preventing that constipated condition of the system which is so invariably productive of numerous bodily discomforts and dangerous and even fatal forms of disease.

